# Effects of inhaled beclometasone dipropionate/formoterol fumarate/glycopyrronium vs. beclometasone dipropionate/formoterol fumarate and placebo on lung hyperinflation and exercise endurance in chronic obstructive pulmonary disease: a randomised controlled trial

**DOI:** 10.1186/s12931-024-02993-x

**Published:** 2024-10-17

**Authors:** Henrik Watz, Anne-Marie Kirsten, Andrea Ludwig-Sengpiel, Matthias Krüll, Robert M. Mroz, George Georges, Guido Varoli, Rémi Charretier, Mauro Cortellini, Andrea Vele, Dmitry Galkin

**Affiliations:** 1 Velocity Clinical Research Grosshansdorf, Airway Research Center North (ARCN), Formerly Pulmonary Research Institute at Lung Clinic Grosshansdorf, Member of the German Center for Lung Research (DZL), Wöhrendamm 80, 22927 Grosshansdorf, Germany; 2 Velocity Clinical Research Lübeck, Formerly KLB Gesundheitsforschung Lübeck GmbH, Lübeck, Germany; 3 Institut für Allergie-und Asthmaforschung Berlin, IAAB, Berlin, Germany; 4 Second Department of Lung Diseases, Lung Cancer and Internal Diseases, Bialystok Medical University, Bialystok, Poland; 5 Global Clinical Development, Chiesi USA Inc, Cary, NC USA; 6 Global Clinical Development, Chiesi Farmaceutici SpA, Parma, Italy; 7 Global Clinical Development, Chiesi SAS, Paris, France

**Keywords:** Dual bronchodilation, Triple therapy, Cycle ergometry, Hyperinflation, Fixed-dose combination

## Abstract

**Background:**

The single-inhaler triple combination of beclometasone dipropionate, formoterol fumarate, and glycopyrronium (BDP/FF/G) is available for maintenance therapy of chronic obstructive pulmonary disease (COPD). Cardinal features of COPD are lung hyperinflation and reduced exercise capacity. TRIFORCE aimed to evaluate the effect of BDP/FF/G on lung hyperinflation and exercise capacity in patients with COPD.

**Methods:**

This double-blind, randomised, active- and placebo-controlled, crossover study recruited adults with COPD aged ≥ 40 years, who were hyperinflated and symptomatic, and were receiving mono- or dual inhaled maintenance COPD therapy. In the three treatment periods, patients were randomised to receive BDP/FF/G, BDP/FF, or placebo, each for 3 weeks, with a 7–10-day washout between treatment periods. Assessments included slow inspiratory spirometry (for resting inspiratory capacity [IC]) and constant work-rate cycle ergometry (for dynamic IC and exercise endurance time). The primary objective was to compare BDP/FF/G and BDP/FF vs. placebo for resting IC at Week 3. Key secondary objectives were to compare BDP/FF/G and BDP/FF vs. placebo for dynamic IC and exercise endurance time during constant work rate cycle ergometry at Week 3.

**Results:**

Of 106 patients randomised, 95 completed the study. Resting IC adjusted mean differences vs. placebo were 315 and 223 mL for BDP/FF/G and BDP/FF, respectively (*p* < 0.001 for both). Adjusted mean differences vs. placebo for the key secondary endpoints were: 245 mL for dynamic IC (*p* < 0.001) and 69.2 s for exercise endurance time (nominal *p* < 0.001) with BDP/FF/G, and 96 mL (*p* = 0.053) and 70.1 s (nominal *p* < 0.001) with BDP/FF. Differences between BDP/FF/G and BDP/FF for resting and dynamic IC were 92 and 149 mL (*p* < 0.01 for both). All three treatments were generally well tolerated, with 27.3%, 25.3% and 19.0% of patients reporting adverse events with BDP/FF/G, BDP/FF and placebo, respectively, all mild or moderate.

**Conclusions:**

In patients with COPD, BDP/FF/G provided significant and clinically relevant improvements vs. placebo and BDP/FF in static and dynamic hyperinflation, with an improvement vs. placebo in exercise endurance.

**Trial registration:**

ClinicalTrials.gov (NCT05097014), registered 27th October 2021.

**Supplementary Information:**

The online version contains supplementary material available at 10.1186/s12931-024-02993-x.

## Background

The triple combination of an inhaled corticosteroid (ICS), a long-acting β_2_-agonist (LABA), and a long-acting muscarinic antagonist (LAMA) is well-established for the maintenance therapy of chronic obstructive pulmonary disease (COPD) [1], with single-inhaler triple therapy associated with improved medication adherence and persistence compared to multiple-inhaler triple therapy [2], a key consideration in COPD management [1]. One such single-inhaler triple therapy is the extrafine formulation of beclometasone dipropionate, formoterol fumarate, and glycopyrronium (BDP/FF/G), the efficacy of which has been evaluated in three large, one-year studies. In TRILOGY, BDP/FF/G provided superior bronchodilation to extrafine formulation dual combination BDP/FF, with an adjusted mean difference of 81 mL in pre-dose forced expiratory volume in 1 s (FEV_1_) at Week 26 (*p* < 0.001), a 23% reduction in the rate of moderate-to-severe exacerbations, and significant improvements in health status [3]. In TRINITY, BDP/FF/G provided superior bronchodilation, a 20% reduction in the rate of moderate-to-severe exacerbations, and significant improvements in health status vs. tiotropium [4]. Finally, in TRIBUTE, BDP/FF/G reduced the rate of moderate-to-severe exacerbations by 15% compared with the fixed-dose LABA/LAMA combination of indacaterol and glycopyrronium [5].

A cardinal feature of COPD is reduced exercise capacity, with symptoms leading to activity limitation, resulting in deconditioning, in turn increasing the impact of symptoms [1, 6, 7]. One key benefit of bronchodilator therapy in COPD is that by reducing lung hyperinflation, exercise capacity can increase, with the effect of mono- and dual bronchodilation on exercise capacity evaluated in a number of previous studies using standardised exercise protocols [8–23]. However, none of the exercise capacity studies published to date have evaluated the effect of adding a LAMA to ICS/LABA. The study reported here, TRIFORCE, aimed to evaluate the effect of BDP/FF/G on lung hyperinflation and exercise capacity in comparison with placebo and BDP/FF in patients with COPD.

## Methods

**Fig. 1:**
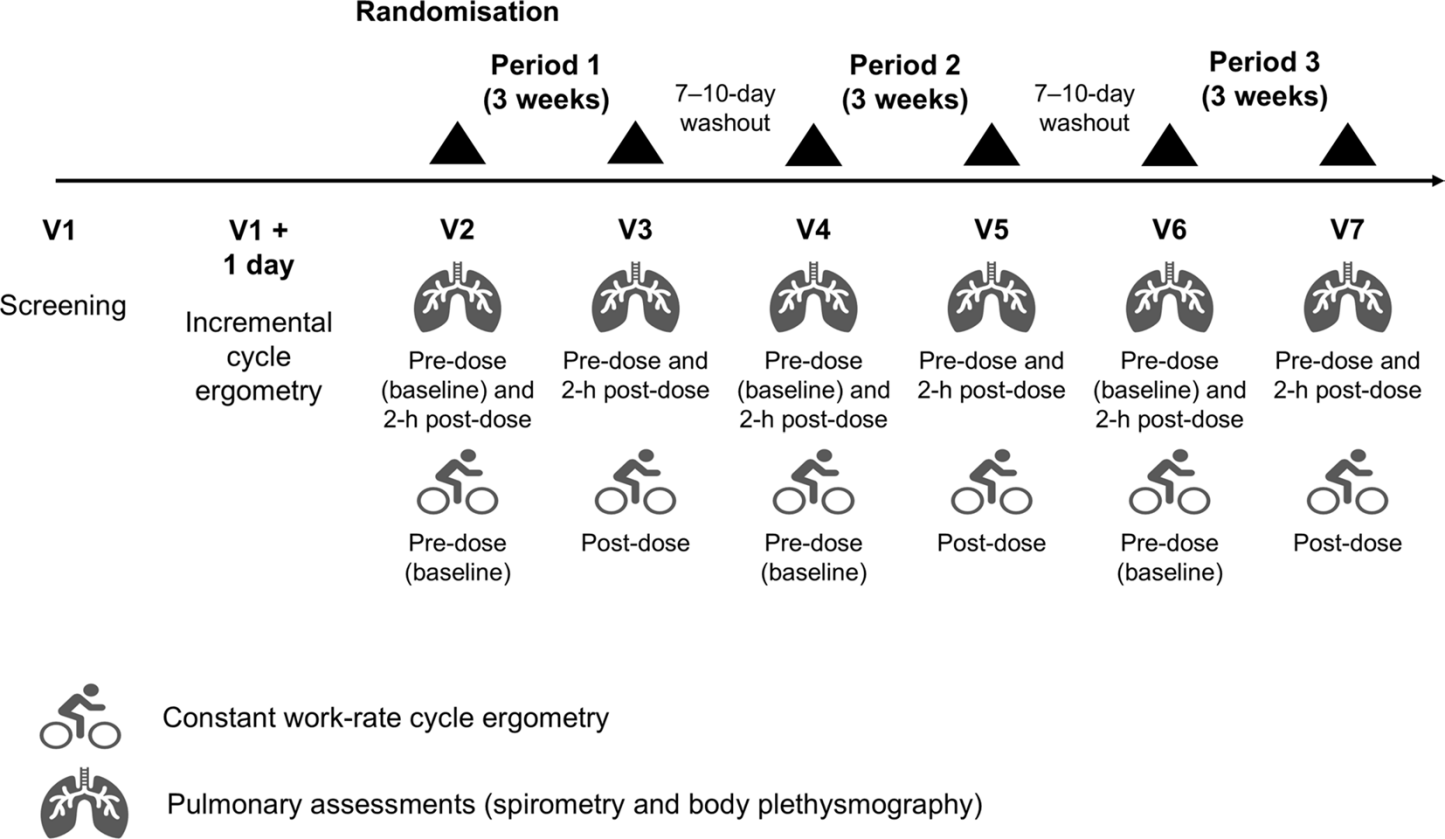
Study design schematic

This was a Phase IV, multinational, multicentre, double-blind, randomised, active- and placebo-controlled, complete block crossover study. After the screening visit, patients completed an incremental exercise test on a computer-driven cycle ergometer to evaluate their peak exercise response (Fig. 1). On a subsequent day they completed a training constant work-rate cycle ergometry test (at 80% of the maximum workload [W_max_] achieved in the incremental exercise test; see the supplement for additional detail). A dedicated cardiopulmonary exercise test manual was provided to each site to standardise the test and minimise inter-operator variability [24, 25]. At the end of a 7–10-day run-in period, eligible patients were randomised to one of six treatment sequences using a balanced-block randomisation scheme generated by the interactive response technology provider. Each sequence comprised three, 3-week treatment periods, with a 7–10-day washout between treatment periods. During the treatment periods, patients received BDP/FF/G 100/6/10 µg per actuation (Trimbow, Chiesi Farmaceutici SpA, Parma, Italy), BDP/FF 100/6 µg per actuation (Foster, Chiesi Farmaceutici SpA, Parma, Italy), or matching placebo, all administered as two inhalations twice daily via identical pressurised metered-dose inhalers. Patients, investigators, site staff and sponsor personnel were blinded to treatment for the duration of the study.

Pre- and 2-h post-dose at the start and end (i.e., after 3 weeks) of each treatment period, patients undertook plethysmography and spirometry assessments. Plethysmography parameters included residual volume (RV), total lung capacity (TLC), RV/TLC ratio, and functional residual capacity (FRC). Spirometry, assessed with standardised spirometry equipment and a central reading service, comprised slow inspiratory manoeuvres to assess resting inspiratory capacity (IC), followed by forced manoeuvres to assess FEV_1_ and forced vital capacity (FVC). Pre-dose on Day 1 of each treatment period and post-dose after 3 weeks (in both cases after forced spirometry), patients completed a constant work-rate cycle ergometry test (at 80% of the W_max_ of the incremental exercise test), during which dyspnoea and muscle fatigue were assessed using a modified Borg scale [26], and IC was measured (prior to initiation, every 2 min during loaded pedalling, and at the end of exercise). Inhaled salbutamol was permitted as rescue medication throughout the study (including during the run-in and washout periods) but not within 6 h prior to any spirometry or cycle ergometry assessment; patients recorded this rescue medication use daily.

All patients provided written informed consent prior to any study-related procedure. The study was approved by the independent ethics committees at each institution (listed in the supplement), and was performed in accordance with the Declaration of Helsinki, and Good Clinical Practice. The study was registered at ClinicalTrials.gov (NCT05097014, registered 27th October 2021). The protocol was amended three times; none of the amendments were substantial or impacted recruitment.

### Participants

Adults ≥ 40 years of age, diagnosed with COPD ≥ 12 months prior to screening, with post-bronchodilator FEV_1_/FVC < 0.7 and FEV_1_ 40–80% predicted were eligible for the study. Participants were hyperinflated (FRC ≥ 120% predicted [9, 11, 14, 17, 18, 20, 22, 23]), symptomatic (modified Medical Research Council dyspnoea scale ≥ 2), and were receiving mono- or dual inhaled maintenance COPD therapy at a stable dose for ≥ 3 months (a regular, scheduled short-acting β_2_-agonist or muscarinic antagonist, alone or in combination, was acceptable), which were to be suspended prior to the screening visit and for the overall study period. Exclusion criteria included known respiratory disorders other than COPD, an abnormal, clinically significant 12-lead electrocardiogram reading that may impact patient safety, unstable concurrent disease or any other disease/condition that may impact the efficacy or safety assessments, and a moderate or severe COPD exacerbation in the previous 3 or 12 months, respectively. The full list of inclusion and exclusion criteria is in the supplement, together with the required wash-out periods prior to the screening visit for maintenance COPD therapy.

### Outcomes

The primary objective was to evaluate the effects of BDP/FF/G and BDP/FF vs. placebo in terms of change from baseline in 2-h post-dose IC, assessed using slow spirometry prior to constant work-rate cycle ergometry (i.e., resting IC) at Week 3 of treatment. The key secondary objectives were to evaluate the effect of BDP/FF/G and BDP/FF vs. placebo in terms of change from baseline at Week 3 in IC at isotime (i.e., dynamic IC) and in exercise endurance time during constant work rate cycle ergometry. Since patients completed two constant work-rate cycle ergometry tests in each treatment period (at the start [baseline], and after 3 weeks), isotime was defined as the shortest exercise endurance time achieved by a patient in either the baseline or Week 3 exercise test, and was derived separately for each treatment period.

Exploratory endpoints included:

- BDP/FF/G vs. BDP/FF comparisons of the primary and key secondary endpoints.
- Change from baseline at Week 3 in:
  - Pre-dose resting IC.
  - Pre-dose FEV_1_.
  - Pre-dose FVC.
  - Pre-dose and 2-h post-dose FRC.
  - Pre-dose and 2-h post-dose RV.
  - Pre-dose and 2-h post-dose RV/TLC ratio.
  - Dyspnoea intensity at isotime (using the modified Borg scale).
- Percentage of rescue medication-free days over the 3-week treatment period.

In addition, *post-hoc* analyses were performed on pre-dose and 2-h post-dose TLC. Safety and tolerability were assessed throughout the study in terms of the occurrence of adverse events, and vital signs, haematology and blood chemistry evaluations.

### Sample size and statistical methods

Assuming a within-patient standard deviation (SD) of 318 mL, using a complete crossover design, 78 evaluable patients would be required to detect a treatment difference of 170 mL in the change from baseline of 2-h post-dose resting IC at Week 3, with 91% power at a two-sided significance level of 0.05. Jointly considering the two comparisons (BDP/FF/G vs. placebo and BDP/FF vs. placebo), the overall power for the primary endpoint would be at least 83%. With a non-evaluable rate of 20%, 102 patients would need to be randomised. This sample size would provide 89% power to detect a difference of 155 mL in dynamic IC at isotime, assuming a within-patient SD of 298 mL, and 84% power to detect a treatment difference of 90 s in exercise endurance time, assuming a within-patient SD of 187 s, both at a two-sided significance of 0.05. The assumptions were based on studies included in a meta-analysis by Di Marco et al. [27].

The primary endpoint was analysed using a linear mixed model assuming an unstructured covariance matrix, including treatment and period as fixed effects, with baseline values for the current period and averaged across all treatment periods as covariates, and patient included as a random effect. Baseline IC was collected pre-dose on Day 1 of each treatment period. The key secondary endpoints were analysed using similar models as the primary endpoint, with baseline dynamic IC and exercise endurance time values taken from the constant workload test conducted pre-dose on Day 1 of each treatment period. Similar models were used to analyse the exploratory and *post-hoc* (TLC) endpoints. Missing data were not imputed.

Type 1 error was controlled for the primary and key secondary endpoints using a hierarchical strategy. Step 1 was the comparison of BDP/FF/G vs. placebo for the primary endpoint; Step 2 was the comparison of BDP/FF vs. placebo for the primary endpoint; Steps 3 and 4 were the comparisons of BDP/FF/G vs. placebo and BDP/FF vs. placebo, respectively, for IC at isotime at Week 3; Steps 5 and 6 were the comparisons of BDP/FF/G vs. placebo and BDP/FF vs. placebo, respectively, for exercise endurance time. Multiplicity was not controlled for the exploratory endpoints.

The efficacy analyses were evaluated in the intention-to-treat (ITT) set, which was all patients who received at least one dose of study medication and who had at least one post-baseline efficacy evaluation. The per-protocol set, which was all patients in the intention-to-treat set without any important protocol deviations, was used for supportive analyses of the primary and key secondary endpoints. The safety set was all patients who received at least one dose of study medication, and was used for all safety analyses.

## Results

The study was conducted between 28th October 2021 and 24th February 2023, at 11 specialist investigative sites in two countries (Germany and Poland; one site in Hungary screened three patients, none of whom were recruited). Of 181 patients screened, 106 were randomised (69 did not meet the inclusion/exclusion criteria, four withdrew consent, and two had a COPD exacerbation), 95 (89.6%) of whom completed the study. Of the 11 who withdrew from the study, eight discontinued due to an adverse event, two had a COPD exacerbation, and one withdrew consent. The majority of recruited patients were male and current smokers, and all were white, with the most common COPD maintenance therapy taken on entry being a LABA/LAMA combination (Table 1).

**Table 1:** Screening demographics and disease characteristics (safety set)

Characteristic	Patients (*N* = 106)
Age, years	65.4 (7.2)
Sex, male	66 (62.3%)
Race, white	106 (100%)
Body-mass index, kg/m^2^	27.13 (4.36)
Smoking status	
Ex-smoker	47 (44.3%)
Current smoker	59 (55.7%)
Time since diagnosis, years	10.39 (7.93)
At least one COPD exacerbation in prior 12 months	16 (15.1%)
COPD maintenance therapy at study entry	
LABA/LAMA	74 (69.8%)
ICS/LABA	14 (13.2%)
LABA	4 (3.8%)
LAMA	5 (4.7%)
SABA	8 (7.5%)
SAMA	1 (0.9%)
Post-bronchodilator FEV_1_	
Absolute, L	1.793 (0.495)
Percent predicted	60.60 (12.10)
GOLD Stage*	
1 (FEV_1_ ≥ 80% predicted)	4 (3.8%)
2 (FEV_1_ < 80% and ≥ 50% predicted)	75 (70.8%)
3 (FEV_1_ < 50% and ≥ 30% predicted)	25 (23.6%)
4 (FEV_1_ < 30% predicted)	1 (0.9%)
Missing	1 (0.9%)
Post-bronchodilator FVC, L	3.470 (0.937)
Post-bronchodilator FEV_1_/FVC	0.526 (0.106)
FRC, L	
Absolute, L	4.745 (0.983)
Percent predicted	144.8 (26.0)
TLC, L	
Absolute, L	6.830 (1.476)
Percent predicted^†^	110.2 (20.1)
RV, L	
Absolute, L	3.609 (1.008)
Percent predicted^†^	156.3 (42.9)
RV/TLC	0.528 (0.112)
Modified medical research council dyspnoea scale	2.2 (0.4)
Constant work-rate cycle ergometry training test	
Reason for termination	
Dyspnoea	27 (25.5%)
Leg	18 (17.0%)
Leg/dyspnoea	55 (51.9%)
Other	6 (5.7%)
Inspiratory capacity, pre-exercise, L	2.205 (0.804)
Modified Borg dyspnoea scale at end exercise	6.86 (2.77)
Exercise endurance time, min	6.12 (2.94)

*The GOLD Stage data are based on the spirometry assessment conducted at the screening visit. If a patient did not meet the spirometry inclusion criterion at this visit (i.e., post-bronchodilator FEV_1_ was not between 40% and 80% predicted), the test could be repeated once prior to the randomisation visit; the patient was randomised only if the inclusion criterion was then met. ^†^The TLC and RV percent predicted data were derived *post-hoc* based on formulae in Stocks and Quanjer [28]. Data are mean (standard deviation) or number (percent). LABA, long-acting β_2_-agonist; LAMA, long-acting muscarinic antagonist; ICS, inhaled corticosteroid; SABA, short-acting β_2_-agonist; SAMA, short-acting muscarinic antagonist; FEV_1_, forced expiratory volume in 1 s; GOLD, Global Initiative for Chronic Obstructive Lung Disease; FVC, forced vital capacity; FRC, functional residual capacity; TLC, total lung capacity; RV, residual volume; RV/TLC, residual volume to total lung capacity ratio

## Outcomes

### Primary and key secondary endpoints

**Fig. 2:**
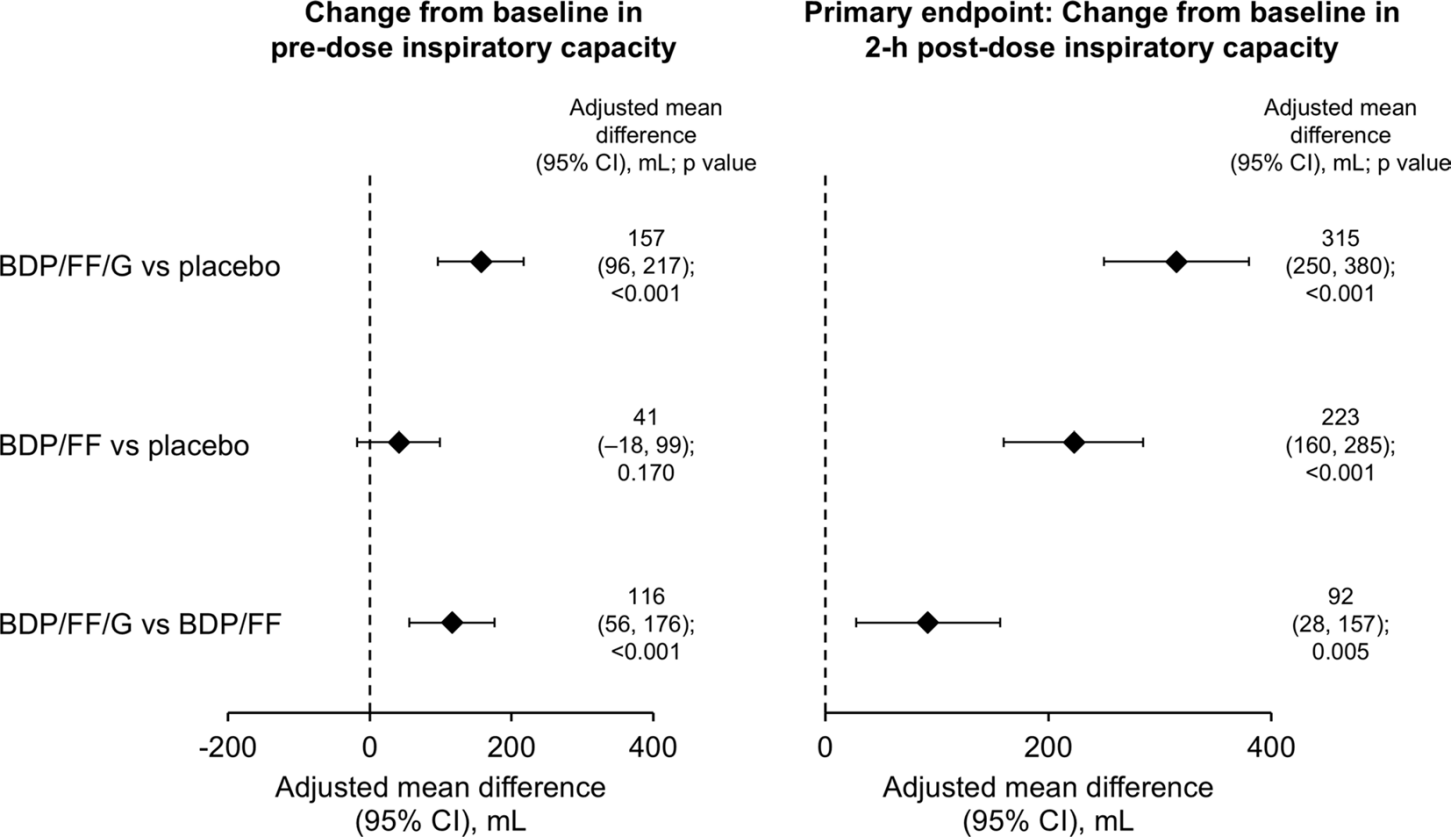
Pre-dose and 2 h post-dose resting inspiratory capacity at Week 3 – adjusted mean differences between treatments (intention-to-treat set). Mean (SD) baseline values were 2.450 (0.693), 2.548 (0.712) and 2.540 (0.733) L for BDP/FF/G, BDP/FF and placebo, respectively. Pre-dose data available from 92, 93 and 93 patients with BDP/FF/G, BDP/FF and placebo, respectively; post-dose data available from 91, 91 and 89 patients, respectively. BDP, beclometasone dipropionate; FF, formoterol fumarate; G, glycopyrronium. The comparisons between BDP/FF/G and BDP/FF (i.e., the last row of the figure) are exploratory endpoints

The primary endpoint was met, with both BDP/FF/G and BDP/FF providing improvements vs. placebo in 2-h post-dose resting IC at Week 3, with adjusted mean differences in the ITT set of 315 and 223 mL, respectively (*p* < 0.001 for both; Fig. 2, with mean values in Supplementary Fig. 1). Results were similar in the supportive analysis on the per-protocol set (differences of 320 and 231 mL, respectively; *p* < 0.001 for both). BDP/FF/G also provided a significant improvement vs. BDP/FF for this endpoint, with a difference in the ITT set of 92 mL (*p* = 0.005).

**Fig. 3:**
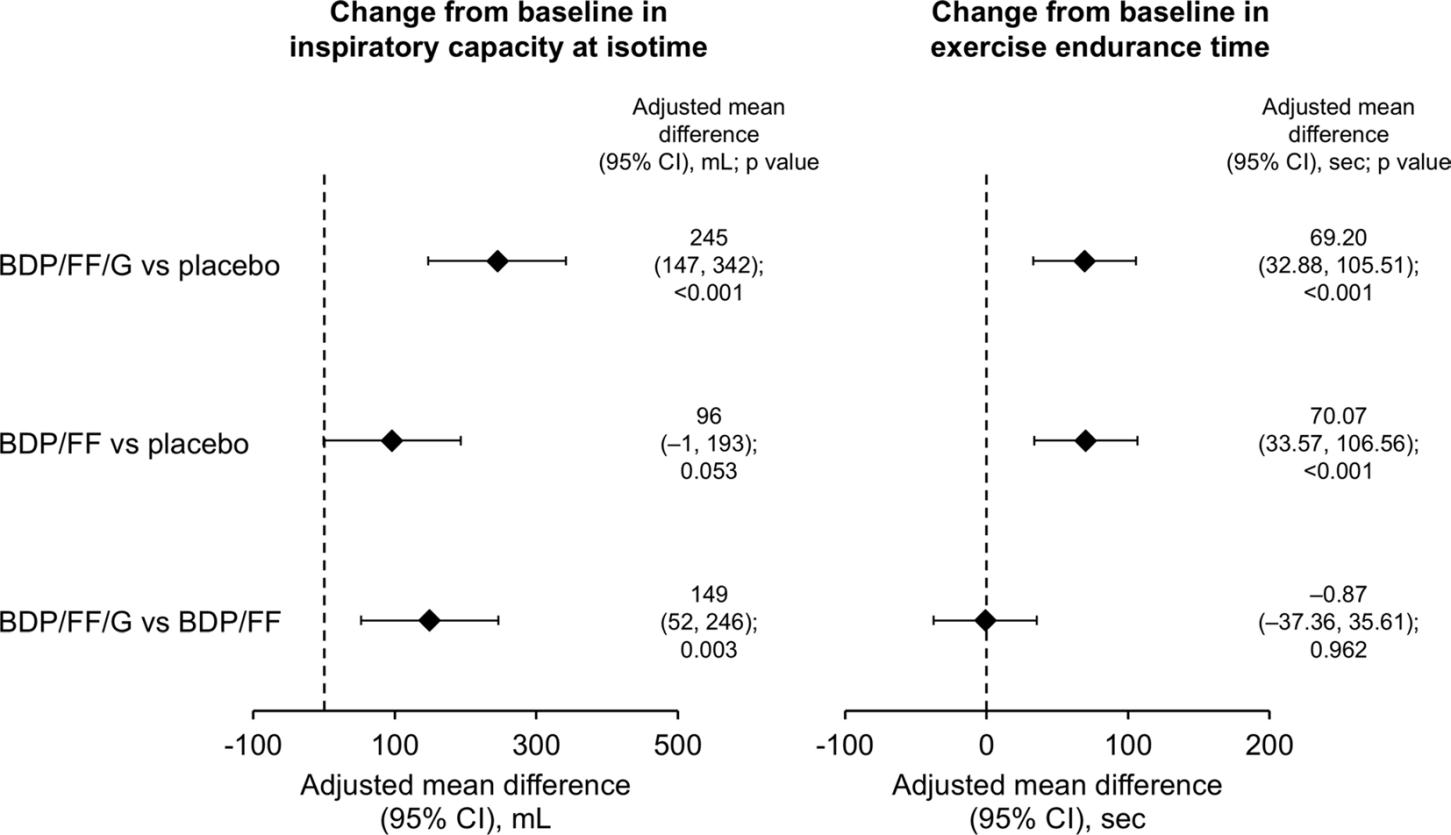
Inspiratory capacity at isotime and exercise endurance time during constant work rate cycle ergometry at Week 3 – adjusted mean differences between treatments (intention-to-treat set). The p values for BDP/FF/G vs. BDP/FF isotime inspiratory capacity and all exercise endurance comparisons are considered nominal only, given Step 4 of the hierarchy (BDP/FF vs. placebo for isotime inspiratory capacity) was not formally achieved. Inspiratory capacity data available from 92, 95 and 92 patients with BDP/FF/G, BDP/FF and placebo, respectively; exercise endurance time data available from 95, 96 and 95 patients, respectively. BDP, beclometasone dipropionate; FF, formoterol fumarate; G, glycopyrronium. The comparisons between BDP/FF/G and BDP/FF (i.e., the last row of the figure) are exploratory endpoints

**Fig. 4:**
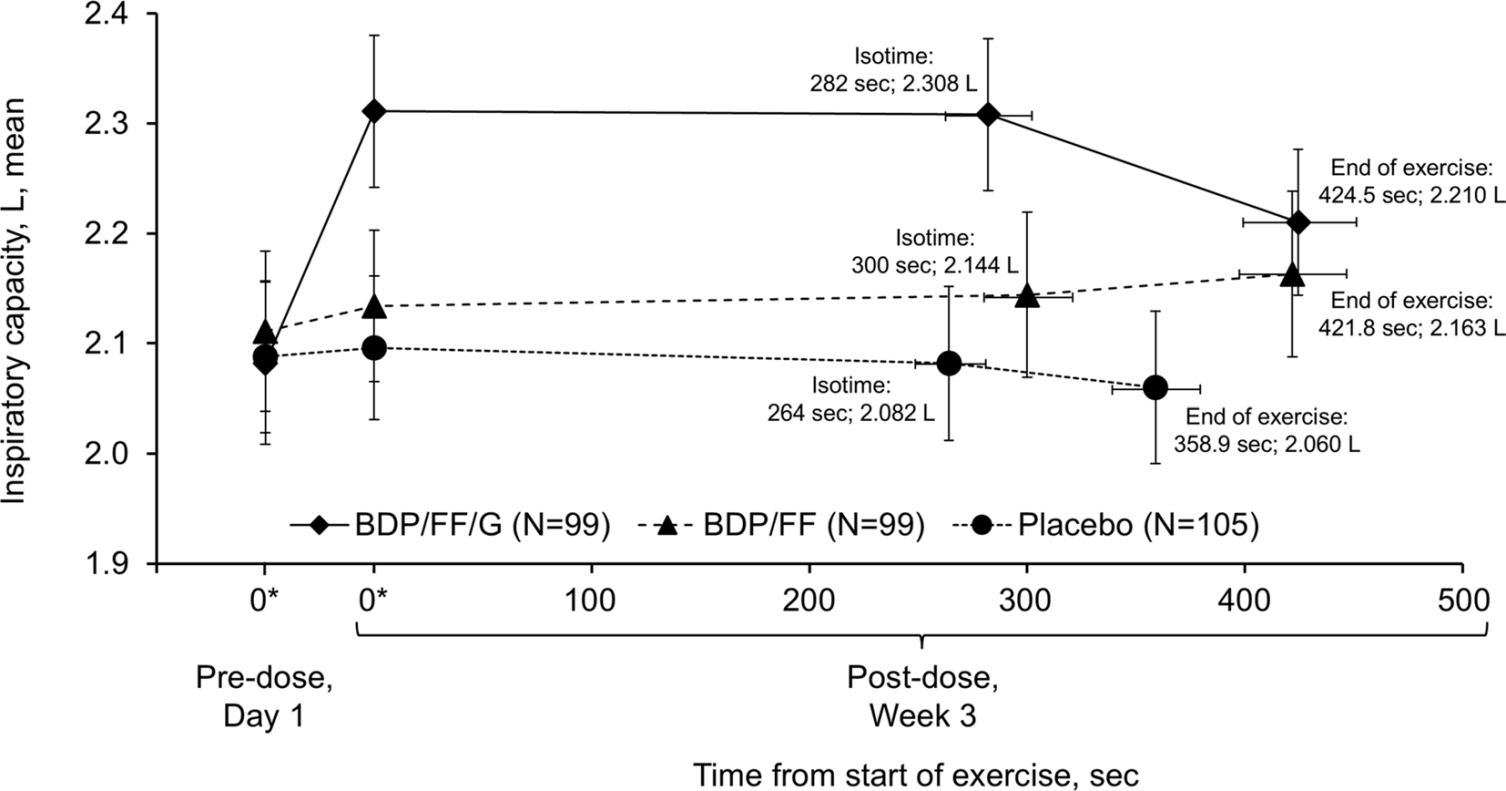
Comparison of mean inspiratory capacity and time from start of exercise during constant work rate cycle ergometry (intention-to-treat set). *Time zero data are resting inspiratory capacity values taken from the constant work rate cycle ergometry test equipment immediately prior to initiation of loaded pedalling (pre-dose on Day 1 and 2-h post-dose at Week 3). BDP, beclometasone dipropionate; FF, formoterol fumarate; G, glycopyrronium

BDP/FF/G also met both of the key secondary endpoints, with adjusted mean improvements vs. placebo in the ITT set of 245 mL for IC at isotime (*p* < 0.001) and 69.2 s for exercise endurance time (nominal *p* < 0.001; Fig. 3, with mean values in Supplementary Figs. 2 and 3). Results were again consistent in the supportive per protocol analyses, with differences of 257 mL and 67.0 s (*p* < 0.001 for both). BDP/FF met the exercise endurance time endpoint, with an adjusted mean improvement vs. placebo in the ITT set of 70.1 s (nominal *p* < 0.001), but not IC at isotime (*p* = 0.053). Of note, in the per protocol set both of these endpoints met the p-value threshold of 0.05 with BDP/FF vs. placebo (101 mL [*p* = 0.046] and 67.5 s [*p* < 0.001]). There was a clinically relevant improvement in IC at isotime with BDP/FF/G compared with BDP/FF of 149 mL (nominal *p* = 0.003), but exercise endurance time was similar with the two active treatments. Figure 4 summarises mean inspiratory capacity vs. exercise times during constant work rate cycle ergometry with the three treatments.

### Exploratory endpoints and post-hoc analyses

**Fig. 5:**
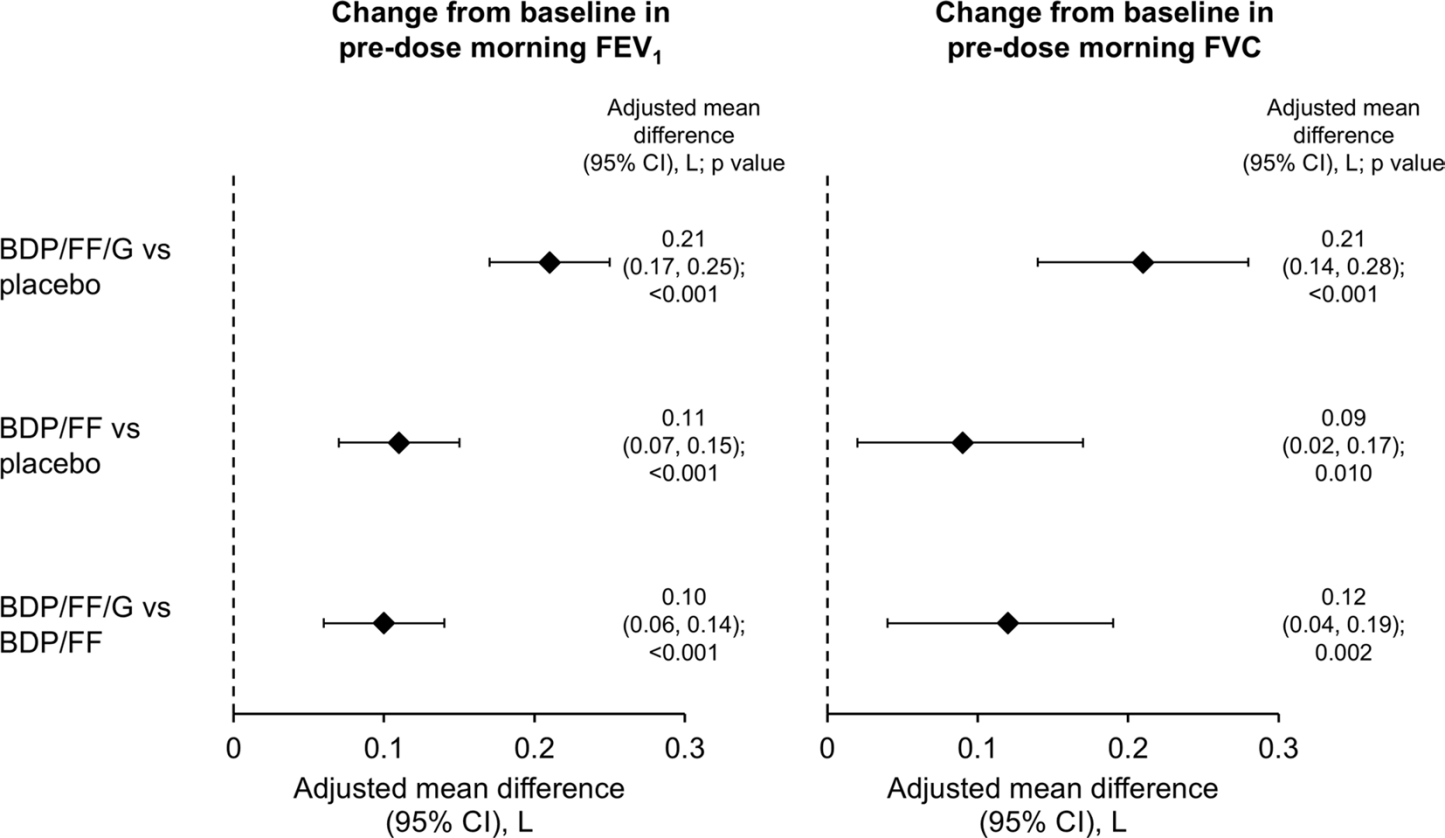
Forced expiratory volume in 1 s (FEV_1_) and forced vital capacity (FVC) assessed pre-dose at Week 3 – adjusted mean differences between treatments (intention-to-treat set). FEV_1_ data available from 98, 98 and 102 patients with BDP/FF/G, BDP/FF and placebo, respectively; FVC data available from 99, 99 and 105 patients, respectively. BDP, beclometasone dipropionate; FF, formoterol fumarate; G, glycopyrronium

BDP/FF/G was statistically superior to placebo and BDP/FF for pre-dose resting IC at Week 3, with clinically relevant differences of 157 and 116 mL, respectively (*p* < 0.001 for both); BDP/FF did not differ from placebo (Fig. 2 and Supplementary Fig. 1). BDP/FF/G and BDP/FF were both statistically superior to placebo for pre-dose FEV_1_ and FVC (*p* < 0.05 for all), with BDP/FF/G superior to BDP/FF (*p* < 0.01; Fig. 5, with the mean values in Supplementary Table 1).

In terms of the plethysmography endpoints, BDP/FF/G and BDP/FF were again consistently statistically superior (i.e., with reductions) to placebo for the Week 3, 2-h post-dose assessments (*p* < 0.05 for all), with BDP/FF/G superior to BDP/FF for RV/TLC (*p* = 0.033) (Supplementary Figs. 4–6 and Supplementary Table 1). For the pre-dose assessments, BDP/FF/G was statistically superior to placebo for FRC and RV (*p* ≤ 0.001), and to BDP/FF for RV (*p* = 0.010), with BDP/FF superior to placebo for FRC (*p* = 0.005). There were no differences in any of the *post-hoc* TLC analyses (Supplementary Fig. 7).

**Fig. 6:**
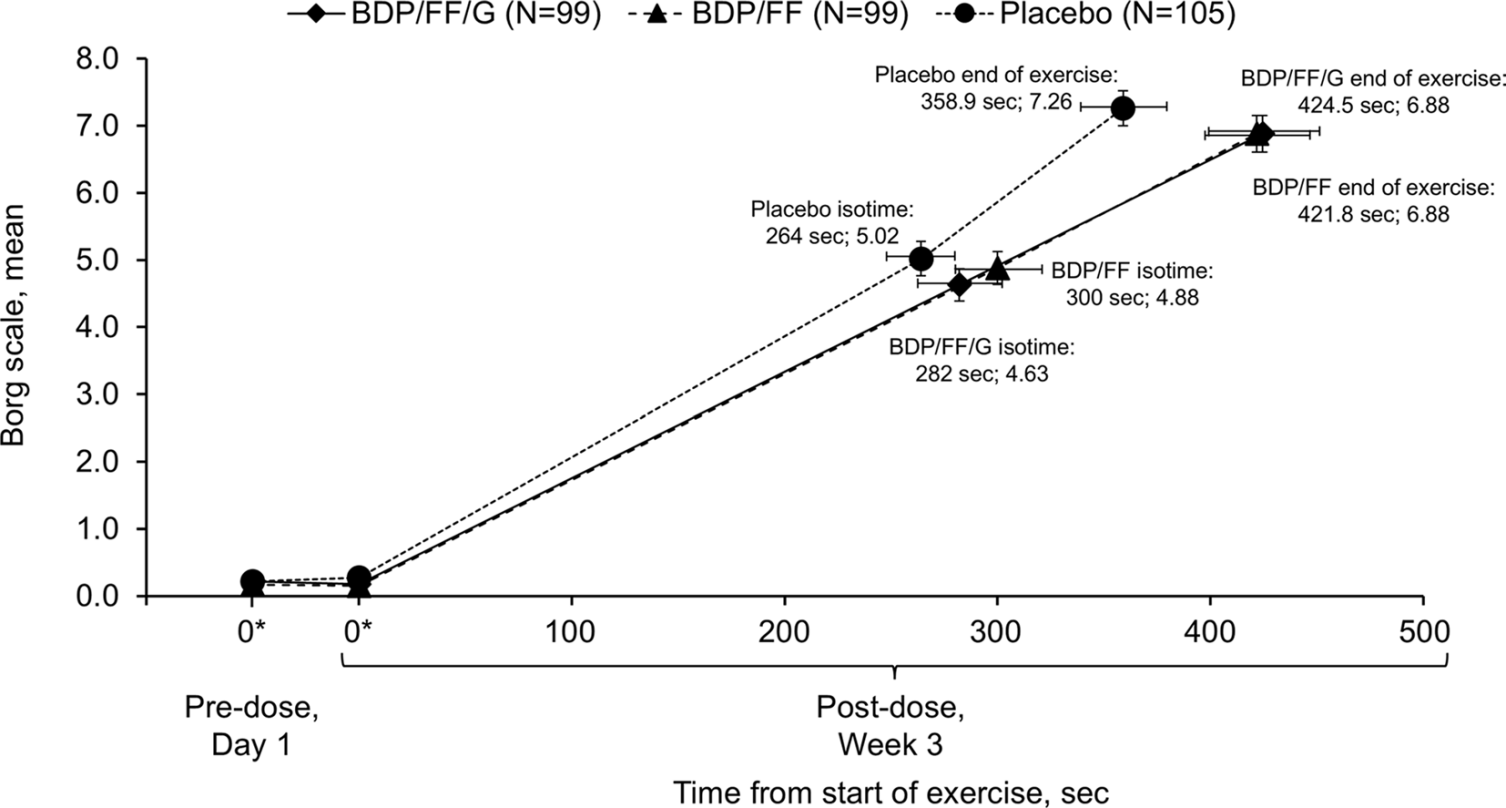
Comparison of mean modified Borg dyspnoea scale data and time from start of exercise during constant work rate cycle ergometry (intention-to-treat set). Note that a lower Borg score indicates less dyspnoea. *Time zero data are values taken from the constant work rate cycle ergometry test equipment immediately prior to initiation of loaded pedalling (pre-dose on Day 1 and 2-h post-dose at Week 3). BDP, beclometasone dipropionate; FF, formoterol fumarate; G, glycopyrronium

The only difference between treatments for modified Borg dyspnoea scale (which was assessed at isotime during exercise) was between BDP/FF/G and placebo, with a statistically significant reduction (i.e., improvement) of 0.43 (*p* = 0.048; Supplementary Fig. 8, with mean Borg dyspnoea vs. exercise time data in Fig. 6). There was an increase in the proportion of rescue-free days with both active treatments vs. placebo (increases of 20.3% [95% CI 13.1%, 27.4%] with BDP/FF/G and 17.2% [10.0%, 24.4%] with BDP/FF; *p* < 0.001 for both), with no difference between actives (3.0 [–4.2, 10.3]; *p* = 0.407 [Supplementary Table 1]).

### Safety

All three treatments were generally well tolerated, with all adverse events being mild or moderate in severity, few considered treatment-related, and none serious (Table 2). The most common adverse event leading to study discontinuation was coronavirus disease 2019 (COVID-19). There were no marked changes from baseline or differences between treatments in vital signs, haematology or blood chemistry assessments.

**Table 2:** Adverse events, overall and most common (occurring in ≥ 2 patients with any treatment; safety set)

	BDP/FF/G (*N* = 99)	BDP/FF (*N* = 99)	Placebo (*N* = 105)
Adverse events	27 (27.3)	25 (25.3)	20 (19.0)
Arthralgia	2 (2.0)	2 (2.0)	2 (1.9)
Chronic obstructive pulmonary disease exacerbation or worsening of symptoms	0	0	4 (3.8)
COVID-19	0	3 (3.0)	4 (3.8)
Cystitis	2 (2.0)	0	0
Diarrhoea	1 (1.0)	2 (2.0)	2 (1.9)
Dysphonia	1 (1.0)	3 (3.0)	0
Dyspnoea	0	0	2 (1.9)
Headache	2 (2.0)	0	1 (1.0)
Nasopharyngitis	4 (4.0)	3 (3.0)	3 (2.9)
Nausea	0	2 (2.0)	1 (1.0)
Oropharyngeal pain	1 (1.0)	2 (2.0)	0
Treatment-related adverse events	1 (1.0)	3 (3.0)	2 (1.9)
Dysphonia	0	2 (2.0)	0
Severe adverse events	0	0	0
Serious adverse events	0	0	0
Adverse event leading to study discontinuation	0	3 (3.0)	7 (6.7)
Chronic obstructive pulmonary disease exacerbation or worsening of symptoms	0	0	3 (2.9)
COVID-19	0	2 (2.0)	3 (2.9)

Data are patients (%). COVID-19, coronavirus disease 2019

## Discussion

The study met the primary objective: BDP/FF/G improved 2-h post-dose resting (static) and dynamic (isotime) hyperinflation, and exercise endurance time vs. placebo. The improvements in IC of 315 and 245 mL exceed the minimum clinically important difference of 140 mL proposed in an official European Respiratory Society (ERS) 2016 statement [24]. Furthermore, although the improvement in exercise endurance time of 69.2 s was lower than the 105 s minimum clinically important difference proposed in the ERS document, it is above the 60 s that is described as the cut-point above which clinical outcomes improve [24], with a difference of 60 s also estimated as the minimum clinically important difference using regression analysis of data from an integrated database that included more than 5000 patients [29]. These improvements vs. placebo were accompanied by improvements in hyperinflation vs. BDP/FF, although not in exercise endurance time, a discrepancy also observed in other exercise studies that evaluated the addition of a second bronchodilator [10, 16], and that might indicate a methodological limitation of this standardised exercise protocol. Furthermore, whereas the improvement in dynamic IC with BDP/FF vs. placebo did not formally reach statistical significance (*p* = 0.053), impacting the statistical hierarchy when controlling Type I error, the improvement in exercise endurance time with BDP/FF vs. placebo was nominally significant (an improvement of 70.1 s; nominal *p* < 0.001), and similar to that with BDP/FF/G vs. placebo (69.2 s; nominal *p* < 0.001). These contrasting results are somewhat surprising, since a reduction in dynamic hyperinflation of this magnitude would be expected to be accompanied by an improvement in exercise capacity. The BDP/FF pre-dose Week 3 resting IC finding is also somewhat surprising, since one would have expected a significant improvement with ICS/LABA vs. placebo, which was not the case in our study. However, modified Borg dyspnoea score at isotime was similarly improved with BDP/FF/G and BDP/FF vs. placebo.

The spirometry and plethysmography data were as expected, with BDP/FF/G providing additional bronchodilation over that provided by BDP/FF, and with a significant improvement in pre-dose FEV_1_ consistent in magnitude with the previous TRILOGY study [3]. Furthermore, BDP/FF/G provided additional improvements over BDP/FF in the various exploratory plethysmography endpoints, although with the BDP/FF/G vs. BDP/FF differences not always reaching statistical significance. In addition, the improvements in RV were consistent with those observed in the TRIFLOW study, a two-period crossover study that compared BDP/FF/G with BDP/FF, both administered for 5 days [30]. All treatments were well tolerated, with no severe or serious adverse events reported, and most of the adverse events that were reported not considered treatment related.

To our knowledge this is the first study to evaluate the effects of inhaled triple therapy on exercise endurance, although a number have compared dual bronchodilation vs. mono-bronchodilation or placebo using constant work rate cycle ergometry [10, 14, 16, 22]. In the BRIGHT study, indacaterol/glycopyrronium was compared with placebo and tiotropium in a three-period crossover study, with each 3-week treatment period separated by a 3-week washout [10]. Both indacaterol/glycopyrronium and tiotropium increased exercise endurance time compared with placebo (by 60 and 66 s, respectively; *p* < 0.01), with no difference between active treatments. In addition, compared with placebo indacaterol/glycopyrronium improved resting IC by 340 mL and dynamic IC at isotime by 320 mL (both *p* < 0.001), with improvements vs. tiotropium of 180 mL in both parameters (both *p* < 0.001). Furthermore, in the parallel-group ACTIVATE study, aclidinium/formoterol was compared with placebo for the first 4 weeks, and a behavioural intervention was added to this pharmacotherapy for an additional 4 weeks, with constant work rate cycle ergometry (at 75% of peak) assessed at Weeks 4 and 8 [22]. The primary endpoint was change from baseline in trough FRC after 4 weeks, which was not met (difference of 125 mL; *p* = 0.069), although it was met in a post-hoc analysis after outlying data from four patients was excluded. Post-dose resting IC and pre-dose FEV_1_ were assessed as additional endpoints, with differences of 293 and 209 mL, respectively (*p* < 0.001 for both). In the constant work rate cycle ergometry, the aclidinium/formoterol–placebo differences at Weeks 4 and 8 were: exercise endurance time 58.9 and 55.2 s (*p* < 0.05 for both); isotime IC 246 and 226 mL (*p* < 0.001). Finally, a series of studies have evaluated the effects of tiotropium/olodaterol on cycle ergometry endpoints [14, 16]. Using data pooled from two replicate, incomplete block crossover studies (MORACTO 1 and 2, comprising three, 6-week treatment periods, separated by 3-week washout periods), tiotropium/olodaterol 5/5 µg (the licensed dose) improved 2-h post-dose resting IC by 245 mL vs. placebo, and by 99 and 101 mL vs. olodaterol and tiotropium, respectively (*p* < 0.001 for all) [16]. Exercise endurance time was significantly prolonged with tiotropium/olodaterol vs. placebo (by 17.3%; *p* < 0.0001) and vs. olodaterol (by 5.6%; *p* < 0.05), although not vs. tiotropium (a non-significant improvement of 1.9%). Dynamic IC at isotime was improved vs. placebo by approximately 250 mL, and by approximately 100 mL vs. tiotropium and olodaterol (*p* < 0.001 for all). Furthermore, Borg score at isotime was lower (i.e., improved) vs. placebo, but not vs. the other actives. In addition, TORRACTO was a 12-week parallel group study that compared tiotropium/olodaterol with placebo [14]. Exercise endurance time was again significantly prolonged with tiotropium/olodaterol 5/5 µg vs. placebo (*p* < 0.05) with dynamic IC at isotime improved vs. placebo by approximately 175 mL (*p* < 0.05), and no difference in Borg at isotime. Taken together, these data suggest that the triple therapy combination of BDP/FF/G is at least as effective on these parameters as the dual bronchodilator combinations indacaterol/glycopyrronium, aclidinium/formoterol and tiotropium/olodaterol.

A number of prior studies have also evaluated the impact of ICS/LABA combinations vs. placebo on exercise capacity. For example, in a three-period crossover study, the effect of 1 week’s treatment with budesonide/formoterol was compared with that of formoterol and placebo in terms of cycle ergometry evaluations [23]. As in the current study, there was an improvement in exercise endurance time with ICS/LABA (budesonide/formoterol) vs. placebo (105 s; *p* < 0.0001), although unlike TRIFORCE this was accompanied by a significant 16% improvement in IC at isotime (*p* < 0.0001). However, in a two-period crossover study that compared the effect of 6 week’s treatment with fluticasone/salmeterol with that of placebo, although fluticasone/salmeterol improved isotime IC by 230 mL vs. placebo (*p* < 0.05), exercise endurance time only increased by a non-significant 1.2 min (*p* = 0.149) [12], very similar to the 70 s difference in TRIFORCE. These contrasting findings clearly illustrate the challenges of conducting cycle ergometry studies.

The main limitation of the study is the 3-week treatment duration, which, although long enough for the three molecules to reach pharmacokinetic steady state, is a relatively short duration for an exercise capacity study. Patients with COPD alter their day-to-day behaviour to avoid symptoms; this results in deconditioning, with these patients then able to achieve less in standardised exercise endurance tests. With the treatment periods being so short, even if patients receive a therapy that effectively prevents the onset of symptoms, they don’t change their daily activities, and therefore don’t overcome the deconditioning. However, lengthening the treatment periods would also have a substantial impact on the overall duration of the study, and may result in higher patient withdrawal, especially during placebo treatment periods.

## Conclusion

In patients with COPD, BDP/FF/G provided statistically significant and clinically relevant improvements vs. placebo in static and dynamic hyperinflation, accompanied by an improvement in exercise endurance time. BDP/FF/G also provided significant and relevant improvements vs. BDP/FF in resting and dynamic hyperinflation, highlighting for the first time the benefit of the addition of a LAMA to ICS/LABA therapy on these parameters. Importantly, both BDP/FF/G and BDP/FF were similarly well tolerated to placebo.

## Electronic supplementary material

Below is the link to the electronic supplementary material.

Supplementary Material 1

## Data Availability

Chiesi commits to sharing with qualified scientific and medical researchers, conducting legitimate research, the anonymised patient-level and study-level data, the clinical protocol and the full clinical study report of Chiesi Farmaceutici SpA-sponsored interventional clinical trials in patients for medicines and indications approved by the European Medicines Agency and/or the US Food and Drug Administration after 1st January 2015, following the approval of any received research proposal and the signature of a Data Sharing Agreement. Chiesi provides access to clinical trial information consistently with the principle of safeguarding commercially confidential information and patient privacy. Other information on Chiesi’s data sharing commitment, access and research request’s approval process are available in the Clinical Trial Transparency section of http://www.chiesi.com/en/research-and-development/.

## Abbreviations

BDP: Beclometasone dipropionate
COPD: Chronic obstructive pulmonary disease
COVID-19: Coronavirus disease 2019
FEV_1_: Forced expiratory volume in 1 s
FF: Formoterol fumarate
FRC: Functional residual capacity
FVC: Forced vital capacity
G: Glycopyrronium
GOLD: Global Initiative for Chronic Obstructive Lung Disease
IC: inspiratory capacity
ICS: Inhaled corticosteroid
LABA: Long-acting β_2_-agonist
LAMA: Long-acting muscarinic antagonist
RV: Residual volume
SD: Standard deviation
TLC: Total lung capacity
W_max_: Maximum workload
