# Supplementary material for: Effects of inhaled beclometasone dipropionate/formoterol fumarate/glycopyrronium vs. beclometasone dipropionate/formoterol fumarate and placebo on lung hyperinflation and exercise endurance in chronic obstructive pulmonary disease: a randomised controlled trial
Source: Respir Res. 2024 Oct 17;25:378. doi: 10.1186/s12931-024-02993-x (PMC11488063; doi:10.1186/s12931-024-02993-x)
Supplement: Supplementary file 1 — Supplementary Material 1 [file 12931_2024_2993_MOESM1_ESM.docx]

# Effects of inhaled beclometasone dipropionate/formoterol fumarate/glycopyrronium vs beclometasone dipropionate/formoterol fumarate and placebo on lung hyperinflation and exercise endurance in chronic obstructive pulmonary disease: A randomised controlled trial

Henrik Watz, Anne-Marie Kirsten, Andrea Ludwig-Sengpiel, Matthias Krüll, Robert M Mroz, George Georges, Guido Varoli, Rémi Charretier, Mauro Cortellini, Andrea Vele, Dmitry Galkin

# Supplement

# Recruiting sites and ethics committees

| **Country** | **Principal investigator** | **Site** | **Ethics committees** |
| --- | --- | --- | --- |
| Germany | Dr Henrik Watz | Pneumologisches Forschungsinstitut an der LungenClinic Grosshansdorf, Großhansdorf | All sites in Germany:  Ethikkommission bei der Ärztekammer SchleswigHolstein, Bad Segeberg, Germany |
|  | Dr med Oliver Kornmann | IKF Pneumologie Frankfurt Institut für klinische Forschung Pneumologie Am Standort IFS - Interdisziplinäres Facharztzentrum, Frankfurt |  |
|  | Dr med. Andrea Ludwig- Sengpiel | KLB Gesundheitsforschung Lübeck GmbH, Lübeck |  |
|  | Dr Stephanie Korn | IKF Pneumologie Mainz Institut für klinische Forschung Pneumologie, Mainz |  |
|  | Dr Olaf Schmidt | KPPK Studienzentrum, Koblenz |  |
|  | Dr Matthias Krüll | Institut für Allergie- und Asthmaforschung Berlin, Berlin |  |
|  | Dr Rainard Fuhr | PAREXEL International GmbH  Early Phase Clinical Unit – Berlin, Berlin |  |
|  | Dr Kai-Michael Beeh | insaf Institut fuer Atemwegsforschung GmbH, Wiesbaden |  |
| Poland | Dr Robert Mroz | Centrum Medycyny Oddechowej Mroz SJ, Bialystok | All sites in Poland:  Komisja Bioetyczna przy Okręgowej Izbie Lekarskiej w Białymstoku, Białystok, Poland |
|  | Dr Iwona Kobielusz-Gembala | Medicome sp.zoo,, Oświęcim |  |
|  | Dr Artur Kwaśniewski | Centrum Diagnostyczno-Terapeutyczne “MEDICUS”, Lubin |  |

# Methods

## Cycle ergometry

The study used each site’s own cycle ergometer. This was required to be electronically braked with a range of 0–600 W, an accuracy of 2% or 3 W above 25 W, and an adjustable seat height and handlebars. This was connected to the metabolic and cardiac monitoring equipment programmed for the specific protocol and with the constant work rate (power output) at pedalling frequencies of 90 rpm.

A laminated card was supplied by the sponsor, which was used for the Borg Scale assessment of breathing and leg discomfort. The patient was asked during exercise to point at the number (0 to 10) while cycling; this was then transferred to the electronic case report form.

### Incremental exercise test

The incremental work rate exercise test was performed with 10 W increments per min until symptom limitation. This was defined as either intolerable shortness of breath or leg fatigue (such that the patient was not able to maintain a cycle frequency of ≥50 rpm). The peak work rate was the highest work-rate that could be maintained for ≥30 s.

### Constant work-rate cycle ergometry tests

These were performed at 80% peak work rate of the incremental exercise test, rounded to the nearest 5 W if the equipment only allowed 5 W steps. As with the incremental test, these tests were continued until symptom limitation, either intolerable shortness of breath or leg fatigue (such that the patient was not able to maintain a cycle frequency of ≥50 rpm), unless stopped by the physician for safety reasons.

## Inclusion criteria

1. A signed and dated written informed consent obtained prior to any study-related procedures.
2. Outpatient population.
3. Male or female subjects ≥40 years at Screening visit.
4. COPD diagnosis for ≥12 months before the Screening visit in accordance with the GOLD 2020 definition.
5. Current or ex-smokers (who quit smoking for ≥6 months prior to Screening Visit) with a smoking history of ≥10 pack-years [pack-years = (number of cigarettes per day x number of years)/20]. *E*-cigarettes smoking cannot be used to calculate pack-year history.
6. A post-bronchodilator FEV_1_/FVC <0.7 within 30 min after 4 puffs (4 x 100 µg) of salbutamol pMDI and a post-bronchodilator FEV_1_ ≥40% and <80% of the predicted normal values.
7. Pre-bronchodilator functional residual capacity (FRC) ≥120% of predicted normal FRC values at Screening visit 1.
8. A score of >2 on the modified Medical Research Council Dyspnoea Scale (mMRC)
   at Visit 1.
9. Subjects on mono- or dual inhaled maintenance COPD treatment at a stable dose for at least 3 months prior to screening.
   **Note:** *examples of inhaled maintenance therapies include LABA, LAMA, ICS+LABA, ICS+LAMA, LABA+LAMA. Subjects receiving scheduled treatments with SABA, SAMA or combination of both are eligible.*
10. A cooperative attitude and ability to correctly use the study inhalers.
11. Female subjects must be either of non-childbearing potential (WONCBP) defined as physiologically incapable of becoming pregnant (i.e. post-menopausal or permanently sterile) or physiologically capable of becoming pregnant (i.e. women of childbearing potential (WOCBP)) fulfilling one of the following criteria:
    1. WOCBP with fertile male partners: they and/or their partner must be willing to use a highly effective birth control method from the signature of the informed consent and until the follow-up contact or
    2. WOCBP with non-fertile male partners (contraception is not required in this case).

**Inclusion criteria assessed prior to randomisation**

1. Constant work-rate cycle ergometry (CWRCE) at Visit 1b: between 2 min and 11 min at 80% of maximum workload.
2. Oxygen saturation (SpO_2_ measured by pulse oximeter) ≥82% during the incremental exercise test (IET) performed in the run-in period.
3. Subjects must be able to complete CWRCE at Visit 1b and then at Visit 2 without requirement for supplemental oxygen.

## Exclusion criteria

1. Pregnant or lactating women.
2. Known respiratory disorders other than COPD which may impact the efficacy of the study drug according to the investigator’s judgment. This can include, but is not limited to, a current diagnosis of asthma, alpha-1 antitrypsin deficiency, active tuberculosis, lung cancer, severe bronchiectasis unrelated to COPD, sarcoidosis, lung fibrosis, pulmonary hypertension and interstitial lung disease.
3. Unstable concurrent disease: e.g. fever, uncontrolled hyperthyroidism, uncontrolled diabetes mellitus or other endocrine disease; significant hepatic impairment; significant renal impairment; uncontrolled gastrointestinal disease (e.g. active peptic ulcer); uncontrolled cardiac disease, uncontrolled neurological disease; uncontrolled haematological disease; uncontrolled autoimmune disorders, or other which may impact the efficacy or the safety of the study drug according to investigator’s judgment.
4. Any other disease/condition which in the opinion of investigator is likely to impact subject’s cardiopulmonary status or the ability to perform functional (exercise) testing during the study.
5. Evidence of symptomatic advanced peripheral artery disease.
6. Moderate (requiring prescriptions of systemic corticosteroids and/or antibiotics) or severe (leading to hospitalisation) COPD exacerbation in the 3 and 12 months, respectively, prior to Screening visit 1 and during the run-in period.
7. Lung transplant or lung volume reduction surgery (subjects with lung volume reduction surgery are excluded if the procedure was performed within 1 year before the Screening visit).
8. Subjects requiring long term (>15 hours a day) oxygen therapy for chronic hypoxemia.
9. Subjects who have clinically severe cardiovascular condition (such as but not limited to unstable ischemic heart disease, New York Heart Association Class IV, left ventricular failure, myocardial infarction in the prior 6 months, not controlled arrhythmia etc.), which may impact the efficacy or the safety of the study drug according to the investigator’s judgement.
10. An abnormal and clinically significant 12-lead electrocardiogram (ECG) which may impact the safety of the subject according to investigator’s judgement. Subjects whose 12-lead ECG shows QTcF >450 msec for males or QTcF >470 msec for females at screening visit are not eligible. Examples of abnormal ECG include atrial fibrillation with rapid ventricular rate >120 bpm, sustained or non-sustained ventricular tachycardia and second-degree heart block Mobitz type II and third-degree heart block.
11. Medical diagnosis of narrow-angle glaucoma, prostatic hypertrophy or bladder neck obstruction that in the opinion of the investigator would prevent use of anticholinergic agents.
12. History of hypersensitivity to M_3_ receptor antagonists, β_2_-agonist, corticosteroids or any of the excipients contained in any of the formulations used in the trial which may raise contra-indications or impact the efficacy of the study drug according to the investigator’s judgement.
13. Clinically significant laboratory abnormalities indicating a significant or unstable concomitant disease which may impact the efficacy or the safety of the study drug according to investigator’s judgement.
14. Subjects with serum potassium levels ≤3.5 mEq/L (or 3.5 mmol/L)
15. Subjects with body mass index <15 or >35 kg/m^2^.
16. Malignancy that has not been in complete remission for at least 1 year or any untreated (e.g. resected for cure) localised carcinomas.
17. History of alcohol abuse and/or substance/drug abuse within 12 months prior to screening visit.
18. Subjects who are mentally or legally incapacitated, or subjects incarcerated as a result of an official or judicial order.
19. Subjects who are in the acute phase of pulmonary rehabilitation program within 1 month before the Screening visit or planning to enrol in the acute phase of such program during the study. Subjects who are in the maintenance phase of a pulmonary rehabilitation program are not excluded.
20. Subjects with contraindications to cardiopulmonary exercise testing, including those whose exercise test is limited by non-respiratory or cardiovascular condition, e.g. by neurologic, orthopaedic, or other disorders.
21. Participation in another clinical trial where investigational drug was received less than 30 days or five half-lives whichever is longer prior to screening visit.

## Washout periods prior to lung function assessments at screening visit for non-permitted concomitant medications

| Inhaled and/or nebulised short-acting β_2_-agonists: | 6 hours |
| --- | --- |
| Inhaled and/or nebulised short acting muscarinic antagonists: | 12 hours |
| Inhaled SABA/SAMA fixed combinations: | 12 hours |
| Inhaled long-acting β_2_-agonists (twice-daily): | 48 hours |
| Inhaled long-acting β_2_-agonists (once-daily): | 72 hours |
| Inhaled and/or nebulised corticosteroids (twice-daily): | 12 hours |
| Inhaled ICS/LABA fixed combinations (twice-daily): | 48 hours |
| Inhaled ICS/LABA fixed combinations (once-daily): | 72 hours |
| Inhaled long acting muscarinic antagonist (LAMA) (once-daily or twice-daily): | 72 hours |
| Inhaled LABA/LAMA (twice-daily): | 72 hours |
| Inhaled LAMA/LABA fixed combinations (once-daily): | 72 hours |
| Leukotriene modifiers: | 72 hours |
| Oral xanthine derivatives: | 48 hours |
| PDE4 inhibitors (e.g. roflumilast): | 7 days |
| Depot corticosteroids: | 8 weeks |
| Oral corticosteroids: | 6 weeks |
| IM/IV corticosteroids: | 6 weeks |
| Antibiotics for (moderate) COPD exacerbation and/or lower respiratory tract infections | 12 weeks |

Note that patients who failed the screening visit were to resume their original maintenance COPD therapy promptly.

# Results

## Supplementary figures

Supplementary Figure 1. Mean resting inspiratory capacity values (intention-to-treat set).


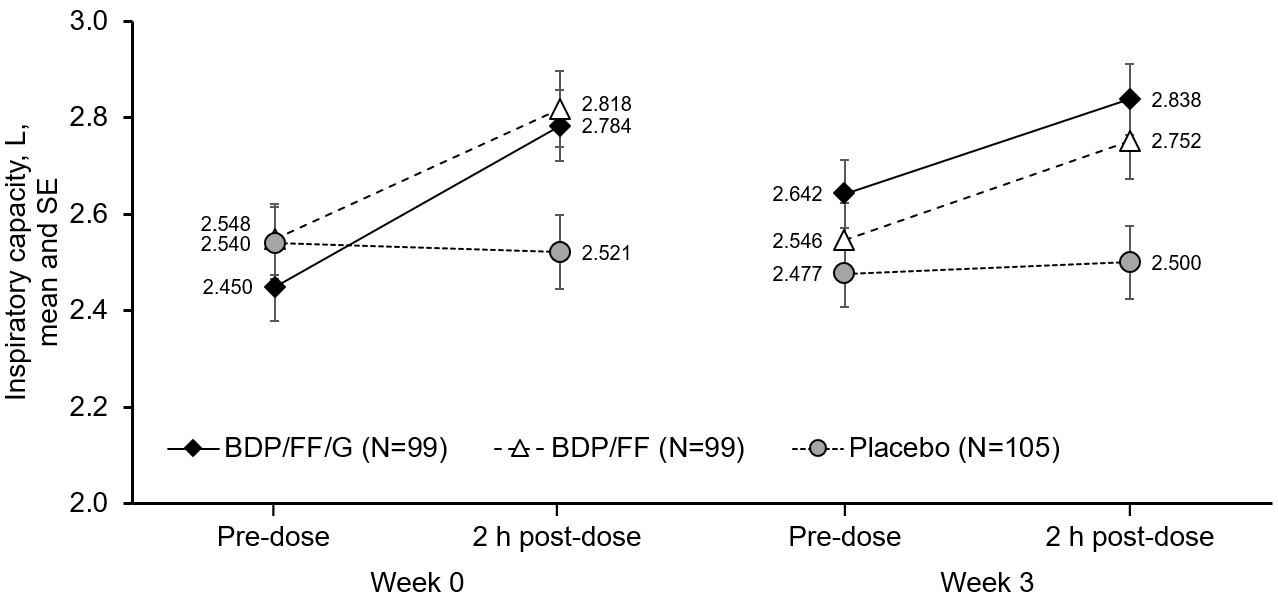


The data are means and standard errors; the standard errors were calculated post-hoc from the standard deviations. BDP, beclometasone dipropionate; FF, formoterol fumarate; G, glycopyrronium.

Supplementary Figure 2. Mean inspiratory capacity values assessed at isotime during constant work rate cycle ergometry (intention-to-treat set).


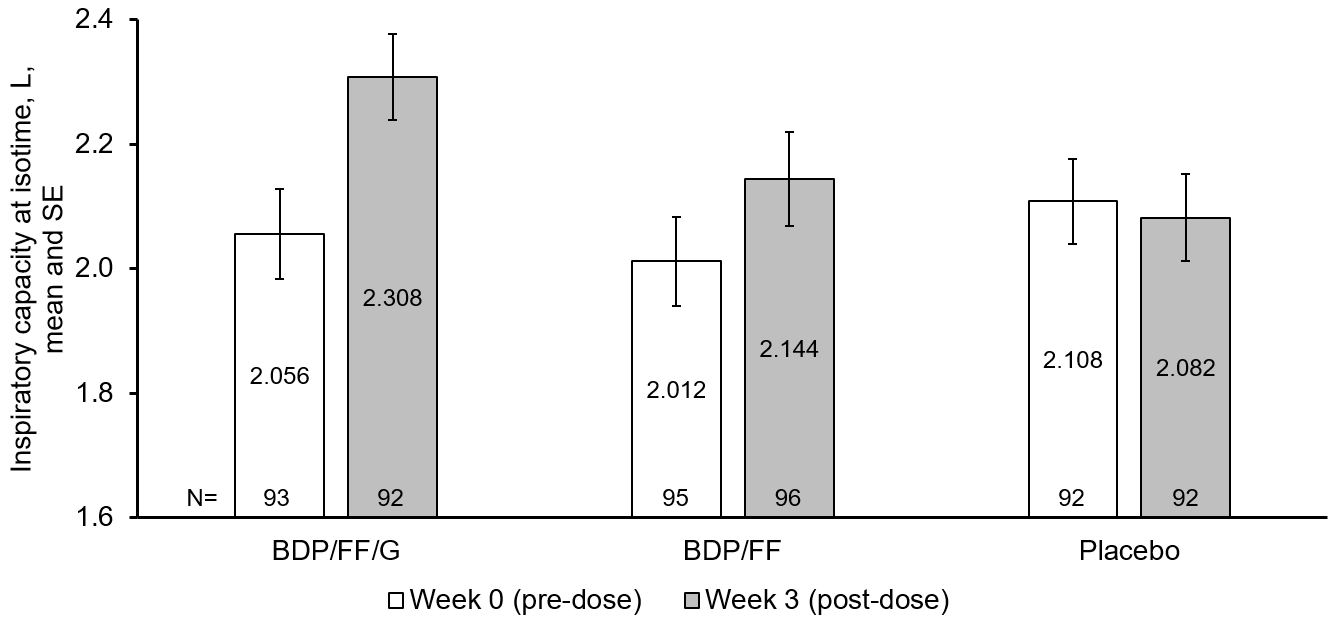


The data are means and standard errors; the standard errors were calculated post-hoc from the standard deviations. BDP, beclometasone dipropionate; FF, formoterol fumarate; G, glycopyrronium.

Supplementary Figure 3. Mean exercise endurance time during constant work rate cycle ergometry (intention-to-treat set).


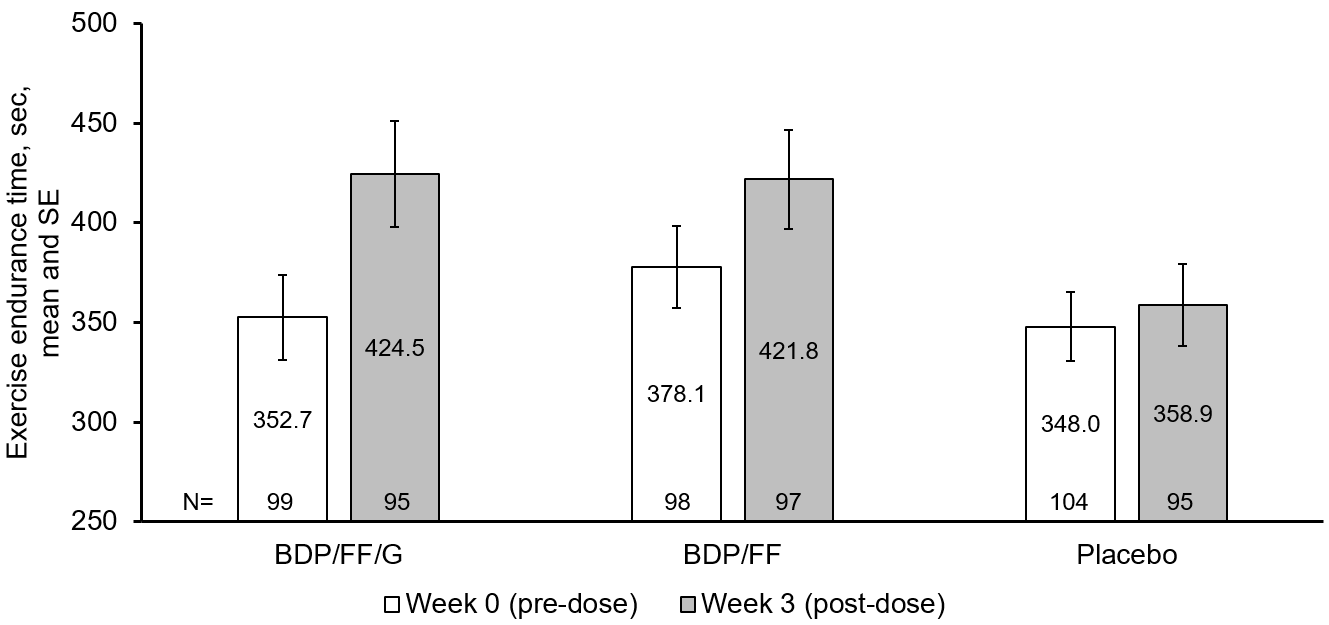


The data are means and standard errors; the standard errors were calculated post-hoc from the standard deviations. BDP, beclometasone dipropionate; FF, formoterol fumarate; G, glycopyrronium.

Supplementary Figure 4. Functional residual capacity (FRC) assessed pre-dose and 2 h post-dose at Week 3 – adjusted mean differences between treatments (intention-to-treat set).


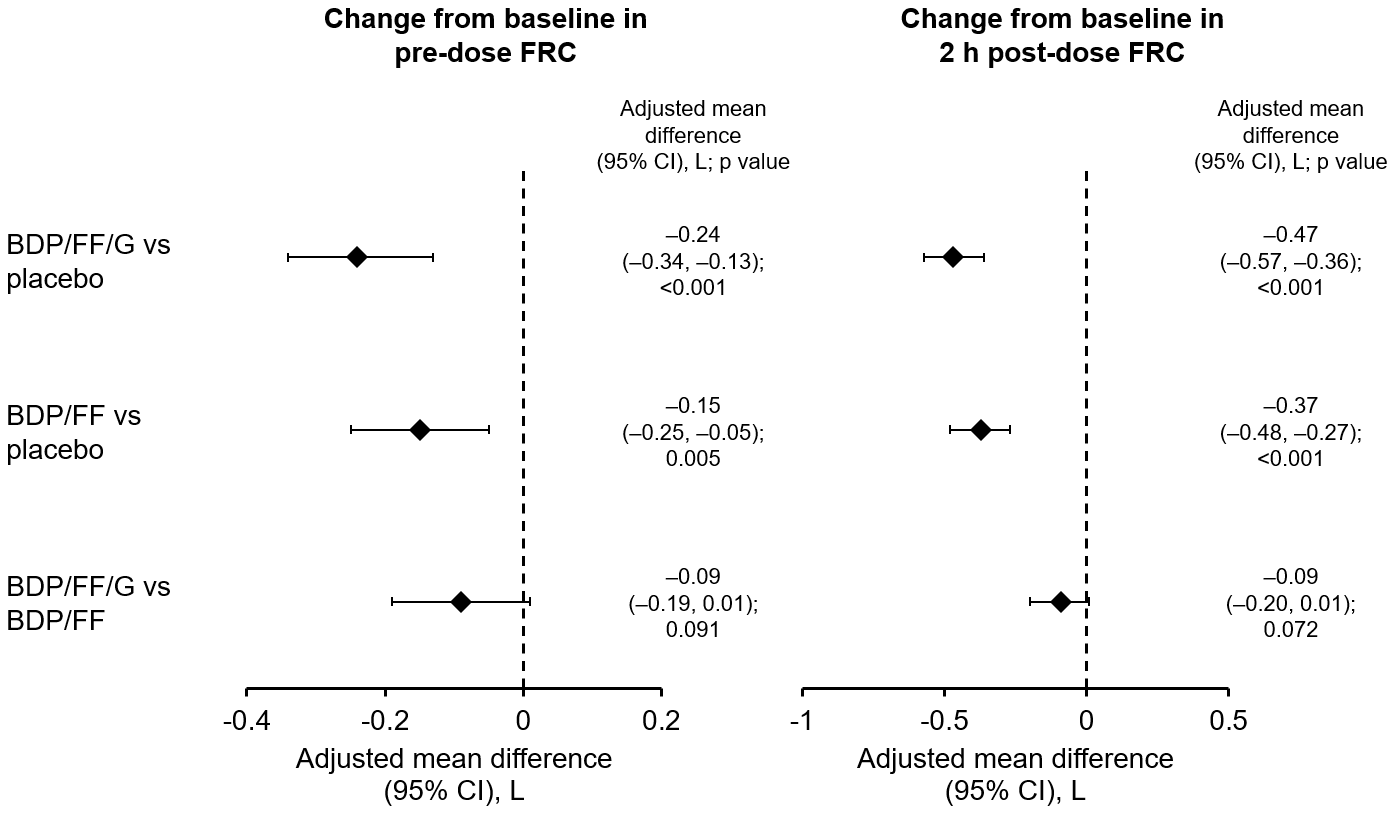


Pre-dose data available from 97, 94 and 94 patients with BDP/FF/G, BDP/FF and placebo, respectively; post-dose data available from 97, 95 and 95 patients, respectively. BDP, beclometasone dipropionate; FF, formoterol fumarate; G, glycopyrronium.

Supplementary Figure 5. Residual volume (RV) assessed pre-dose and 2 h post-dose at Week 3 – adjusted mean differences between treatments (intention-to-treat set).


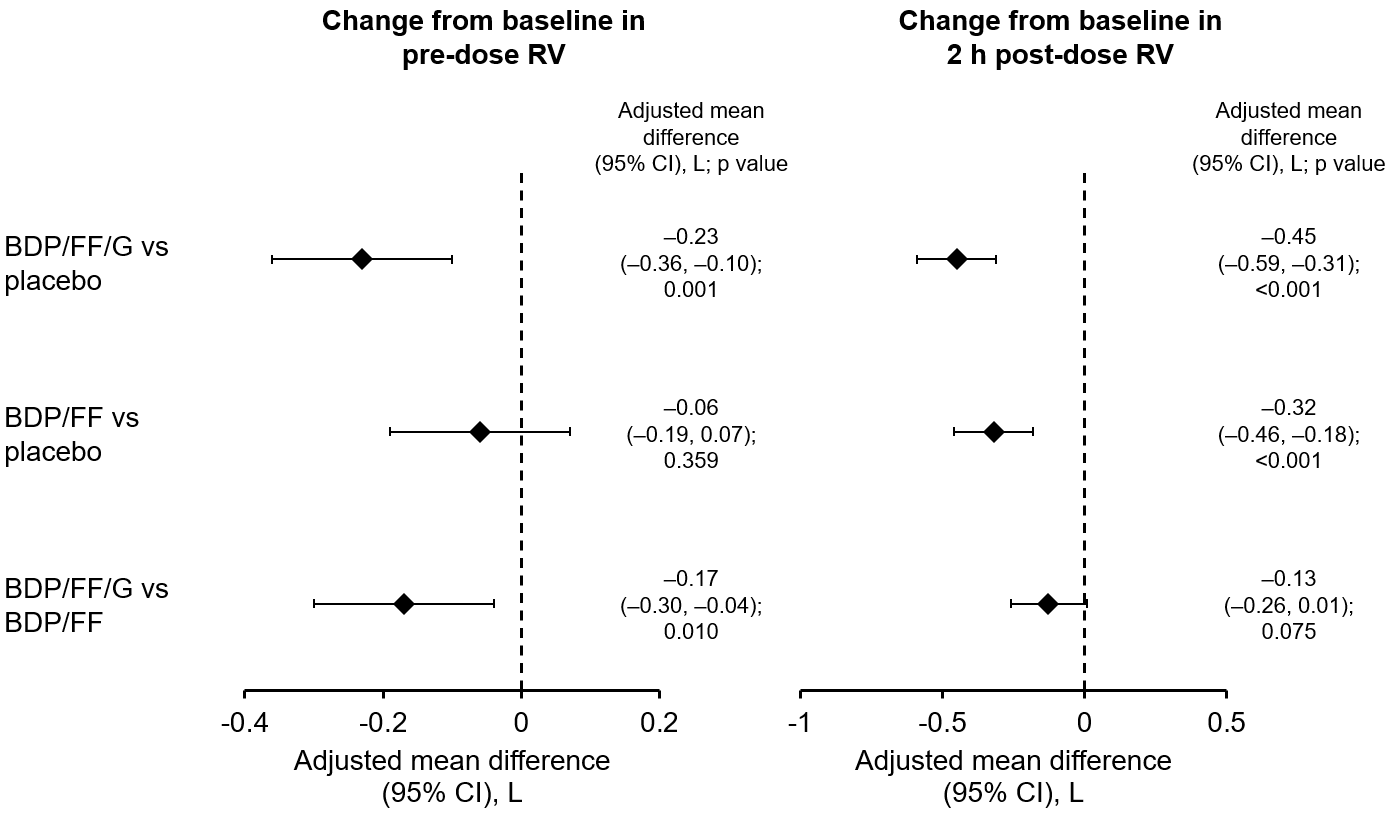


Pre-dose data available from 97, 94 and 94 patients with BDP/FF/G, BDP/FF and placebo, respectively; post-dose data available from 97, 95 and 95 patients, respectively. BDP, beclometasone dipropionate; FF, formoterol fumarate; G, glycopyrronium.

Supplementary Figure 6. Ratio of residual volume to total lung capacity (RV/TLC) assessed pre-dose and 2 h post-dose at Week 3 – adjusted mean differences between treatments (intention-to-treat set).


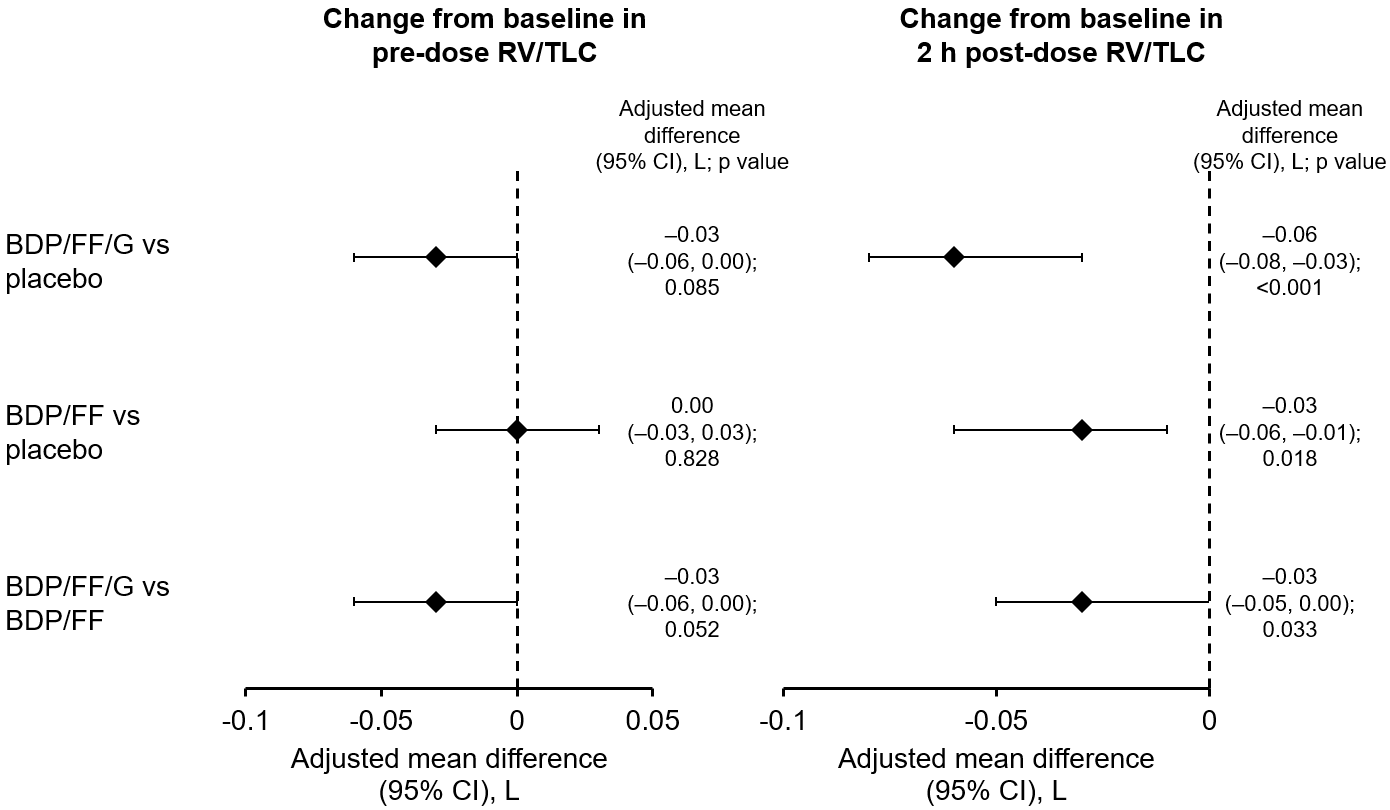


Pre-dose data available from 97, 94 and 94 patients with BDP/FF/G, BDP/FF and placebo, respectively; post-dose data available from 97, 95 and 95 patients, respectively. BDP, beclometasone dipropionate; FF, formoterol fumarate; G, glycopyrronium.

Supplementary Figure 7. Total lung capacity (TLC) assessed pre-dose and 2 h post-dose at Week 3 – adjusted mean differences between treatments (intention-to-treat set; post-hoc analyses).


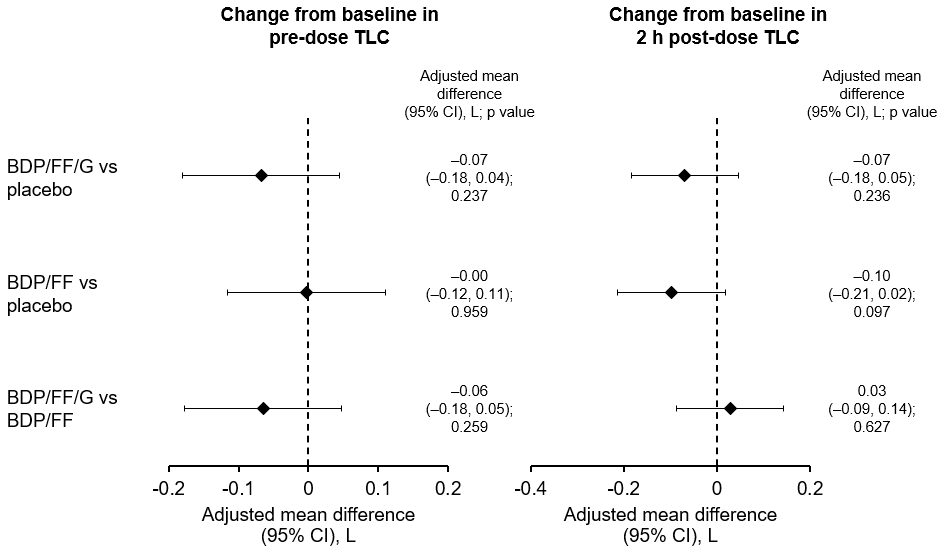


Pre-dose data available from 97, 95 and 95 patients with BDP/FF/G, BDP/FF and placebo, respectively; post-dose data available from 97, 96 and 96 patients, respectively. BDP, beclometasone dipropionate; FF, formoterol fumarate; G, glycopyrronium.

Supplementary Figure 8. Modified Borg dyspnoea score at isotime assessed at Week 3 – adjusted mean differences between treatments (intention-to-treat set).


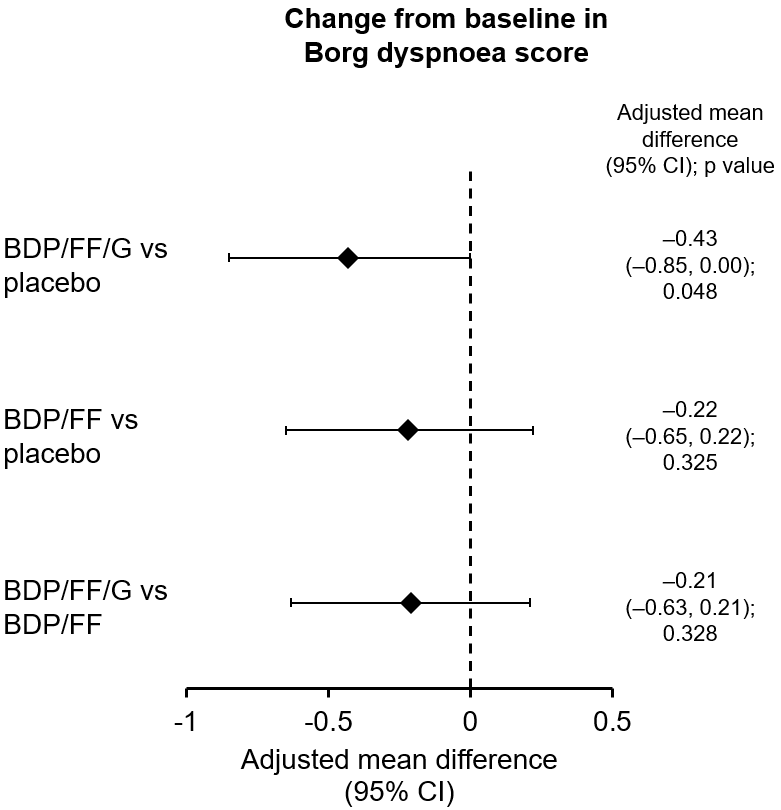


Data available from 93, 96 and 92 patients with BDP/FF/G, BDP/FF and placebo, respectively. BDP, beclometasone dipropionate; FF, formoterol fumarate; G, glycopyrronium.

## Supplementary table

Supplementary Table 1. Mean values for exploratory endpoints.

|  | **Baseline** | **Week 3, pre-dose** | **Week 3, 2-h post-dose** |
| --- | --- | --- | --- |
| Morning forced expiratory volume in 1 sec, L | |  |  |
| BDP/FF/G | 1.591 (0.536)  (N=99) | 1.775 (0.532)  (N=98) | – |
| BDP/FF | 1.636 (0.528) (N=99) | 1.686 (0.542) (N=98) | – |
| Placebo | 1.600 (0.551) (N=105) | 1.547 (0.539) (N=102) | – |
| Morning forced vital capacity, L | |  |  |
| BDP/FF/G | 3.267 (0.949)  (N=99) | 3.463 (0.924)  (N=98) | – |
| BDP/FF | 3.331 (0.933) (N=99) | 3.351 (0.943) (N=98) | – |
| Placebo | 3.287 (0.985) (N=105) | 3.236 (0.987) (N=102) | – |
| Functional residual capacity, L | |  |  |
| BDP/FF/G | 4.797 (1.143)  (N=99) | 4.555 (1.101)  (N=97) | 4.238 (0.985)  (N=97) |
| BDP/FF | 4.829 (1.112) (N=98) | 4.656 (1.069) (N=95) | 4.363 (0.967) (N=96) |
| Placebo | 4.775 (0.979) (N=103) | 4.756 (1.084) (N=95) | 4.670 (1.116) (N=96) |
| Residual volume, L |  |  |  |
| BDP/FF/G | 3.681 (1.139)  (N=99) | 3.379 (1.026) (N=97) | 3.106 (0.823)  (N=97) |
| BDP/FF | 3.630 (1.188) (N=98) | 3.514 (1.070) (N=95) | 3.179 (0.964) (N=96) |
| Placebo | 3.600 (1.019) (N=103) | 3.593 (1.043) (N=95) | 3.508 (1.094) (N=96) |
| Total lung capacity, L |  |  |  |
| BDP/FF/G | 6.889 (1.563) (N=99) | 6.691 (1.493) (N=97) | 6.699 (1.383) (N=97) |
| BDP/FF | 6.814 (1.567) (N=98) | 6.725 (1.508) (N=95) | 6.608 (1.406) (N=96) |
| Placebo | 6.792 (1.489) (N=103) | 6.721 (1.428) (N=95) | 6.697 (1.484) (N=96) |
| Residual volume to total lung capacity ratio | |  |  |
| BDP/FF/G | 0.532 (0.107)  (N=99) | 0.505 (0.119) (N=97) | 0.465 (0.095)  (N=97) |
| BDP/FF | 0.528 (0.134) (N=98) | 0.529 (0.179) (N=95) | 0.485 (0.160) (N=96) |
| Placebo | 0.528 (0.116) (N=103) | 0.532 (0.116) (N=95) | 0.520 (0.116) (N=95) |
| COPD Assessment Test total score | |  |  |
| BDP/FF/G | 17.2 (6.3)  (N=99) | 15.8 (6.3)  (N=99) | – |
| BDP/FF | 17.1 (6.5) (N=99) | 15.7 (6.1) (N=98) | – |
| Placebo | 16.7 (5.8) (N=105) | 18.2 (6.1) (N=103) | – |
| Modified Borg dyspnoea scale score at isotime | |  |  |
| BDP/FF/G | 5.04 (2.45)  (N=93) | 4.63 (2.31)  (N=93) | – |
| BDP/FF | 5.38 (2.65) (N=93) | 4.88 (2.38) (N=96) | – |
| Placebo | 4.80 (2.57) (N=93) | 5.02 (2.49) (N=92) | – |
| Rescue medication free days, % | |  |  |
| BDP/FF/G | 40.8 (41.3)  (N=91) | 71.9 (34.9)  (N=97) |  |
| BDP/FF | 41.0 (41.2) (N=91) | 67.9 (37.0) (N=98) |  |
| Placebo | 39.7 (40.9) (N=95) | 50.0 (42.5) (N=102) |  |

Data are mean (SD); rescue medication data were assessed over the 3-week baseline and treatment periods. BDP, beclometasone dipropionate; FF, formoterol fumarate; G, glycopyrronium.
